# Asthma, Rhinoconjunctivitis, Eczema, and the Association with Perinatal Anthropometric Factors in Vietnamese Children

**DOI:** 10.1038/s41598-019-39658-5

**Published:** 2019-02-25

**Authors:** Michiko Toizumi, Masahiro Hashizume, Hien Anh T. Nguyen, Michio Yasunami, Noriko Kitamura, Chihiro Iwasaki, Mizuki Takegata, Hiroyuki Moriuchi, Duc Anh Dang, Koya Ariyoshi, Lay-Myint Yoshida

**Affiliations:** 1 Department of Pediatric Infectious Diseases, Institute of Tropical Medicine, Nagasaki University, Nagasaki, Japan; 2 Department of Global Health, School of Tropical Medicine and Global Health, Nagasaki University, Nagasaki, Japan; 3 Department of Bacteriology, National Institute of Hygiene and Epidemiology, Hanoi, Vietnam; 4 Science Life Institute, Saga-ken Medical Centre Koseikan, Saga, Japan; 5 Department of Clinical Medicine, Institute of Tropical Medicine, Nagasaki University, Nagasaki, Japan; 6 Graduate School of Biomedical Sciences, Nagasaki University, Nagasaki, Japan

## Abstract

Few studies have investigated possible causative and protective factors associated with allergic diseases in resource-limited countries, Southeast Asia. We estimated the current prevalence of asthma, rhinoconjunctivitis, and eczema among 6-year-old children, and identified anthropometric factors associated with asthma, rhinoconjunctivitis and eczema, in South-Central Vietnam. A birth cohort study recruited 1,999 children born at a provincial hospital in Nha Trang, Vietnam between May 2009 and May 2010. A 6-year follow-up survey was conducted where clinical, familial, and environmental information was collected by interviewing caregivers using a standardized form based on the International Study of Asthma and Allergies in Childhood, Phase Three Core and Environmental Questionnaire for 6–7-year-old children. The odds ratios of asthma, rhinoconjunctivitis, and eczema for anthropometric factors were estimated using logistic regression analysis. In total, 1202 children participated in the follow-up survey. The proportions of children who had current asthma, rhinoconjunctivitis, and eczema were 5.1% (95% confidence interval [CI] 3.9–6.5%), 11.5% (9.7–13.4%), and 6.7% (5.3–8.2%), respectively. Low birthweight (adjusted odds ratio 5.12, 95% CI 1.92–13.64) was independently associated with increased risk of eczema. Further studies are necessary to understand the involved mechanism.

## Introduction

Many studies have shown that the prevalence of asthma, rhinoconjunctivitis, and eczema has increased in the world and the prevalence has large geographical variations. They were more prevalent in urbanized societies than in rural or developing countries^1–4^.

Low birthweight has been shown to be associated with wheezing disorders or asthma in childhood and adulthood life^5–7^. A meta-analysis including 37 studies comprising 1,712,737 participants suggested that low birthweight was an independent risk factor for wheezing disorders, although there was substantial heterogeneity among the risk estimates^8^. Also, growth restriction followed by rapid catch-up growth was shown to be related to impaired lung function and asthma in some studies^9–12^. Several studies have examined the relationship between anthropometric parameters at birth and atopic dermatitis or eczema. Some of these studies found a positive correlation between birthweight and atopic eczema^13,14^, whereas others found no such association^15,16^. Panduru *et al*. suggested that low birthweight was a protective factor for the occurrence of atopic dermatitis in their meta-analysis of 10 studies that discussed birthweight and atopic dermatitis^17^. These results indicate that the aetiology of asthma and atopic eczema involved fetus-specific mechanisms^9^. Most studies of low birth weight and allergic rhinitis found no association^15,18,19^ but one study showed positive association^20^ between them.

Vietnam is undergoing rapid economic development and urbanization. Chronic childhood diseases such as asthma, rhinoconjunctivitis and eczema may become a major health priority in Vietnam similar to other countries in Asia^21^. However, there are few studies of asthma, rhinoconjunctivitis and eczema using standardized protocols to investigate possible causative and protective factors in Vietnam^22–24^. Using perinatal parameters as potential risk factors for later life problems is particularly difficult in Vietnam because of the lack of precise information at birth. However, our birth cohort study has provided accurate perinatal information for analysis.

In this study, we followed up children enrolled into our birth cohort study in Nha Trang, South-Central Vietnam and asked their allergic status at age 6 using a standardized questionnaire. Our objectives were, first, to estimate the prevalence of current asthma, rhinoconjunctivitis, and eczema among 6-year-old children in South-Central Vietnam, and second, to evaluate the association between low birthweight (primary exposure) and asthma, rhinoconjunctivitis, and eczema.

## Results

### Characteristics of participants

**Figure 1:**
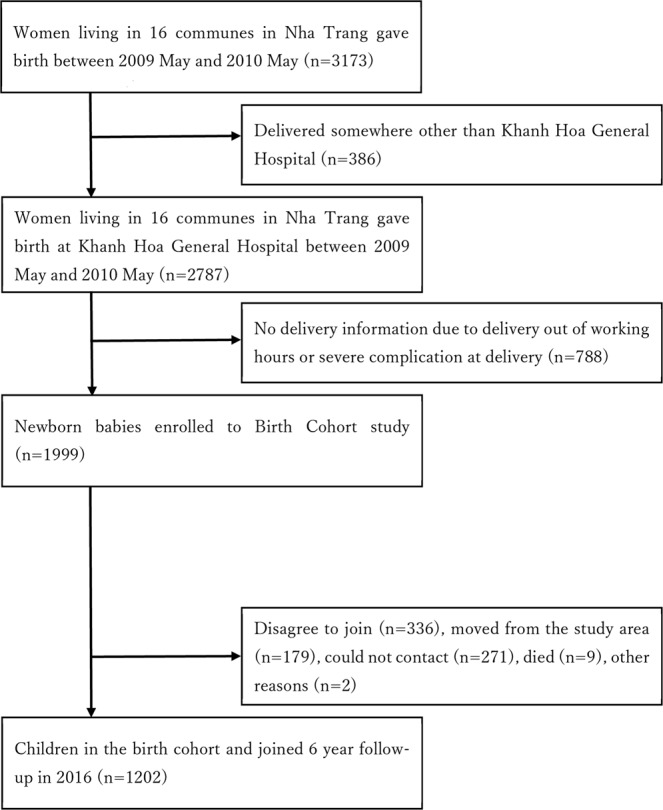
Details of the enrollment and follow-up of the study participants.

A total of 1999 babies were enrolled to the birth cohort. Among them, 1202 (60.1%) children participated in the 6-year follow-up survey in 2016. Figure 1 shows details of the recruitment and follow-up processes. Sex, mode of delivery, and proportions of low birthweight and preterm born data were similar for the children who participated in the follow-up survey and those who did not (p = 0.3, p = 0.3, p = 0.7, and p = 0.2 in chi-square test, respectively). The characteristics of the children who participated in the follow-up survey are shown in Table 1.

**Table 1:** Characteristics of the children who participated in the six-year follow-up survey.

Characteristics (n = 1202)			Number (%)/ mean (standard deviation)
**Demographics**			
	Sex	Male	610 (50.8)
		Female	592 (49.3)
	Age (years old)		6.7 (0.2)
	Body weight (kg)		25.9 (5.8)
	Body weight		
		25 kg or more	568 (47.3)
		less than 25 kg	634 (52.8)
	Body height (cm)		119.7 (5.9)
**Perinatal information**			
	Mode of delivery		
		Vaginal	688 (57.2)
		Caesarean section	514 (42.8)
	Birthweight (g)		3267.9 (411.5)
	Birthweight		
		>=2500 g	1175 (97.8)
		<2500 g	27 (2.3)
	Gestational age at birth (weeks)		39.6 (1.2)
	Gestational age at birth		
		>=37 weeks	1166 (97.0)
		<37 weeks	36 (3.0)
	Light-for-dates		129 (10.7)
**Family history**			
	Mother’s age at child’s birth		
		24 years or less	312 (26.0)
		25–34 years	728 (60.6)
		35 years or more	162 (13.5)
(n = 1201)	Maternal history of asthma and allergic diseases		
		Yes	96 (8.0)
		No	1105 (92.0)
(n = 1201)	Maternal history of asthma		
		Yes	12 (1.0)
		No	1189 (99.0)
(n = 1201)	Maternal history of atopic eczema		
		Yes	13 (1.1)
		No	1188 (98.9)
(n = 1201)	Maternal history of rhinitis		
		Yes	76 (6.3)
		No	1125 (93.7)
(n = 1191)	Paternal history of asthma and allergic diseases		
		Yes	47 (4.0)
		No	1144 (96.1)
(n = 1191)	Paternal history of asthma		
		Yes	9 (0.8)
		No	1182 (99.2)
(n = 1191)	Paternal history of atopic eczema		
		Yes	5 (0.4)
		No	1186 (99.6)
(n = 1191)	Paternal history of rhinitis		
		Yes	33 (2.8)
		No	1158 (97.2)
(n = 1201)	Maternal education level		
		No or primary school	152 (12.7)
		Secondary school	403 (33.5)
		High school	399 (33.2)
		College or university	248 (20.6)
**Environmental status**			
	Number of siblisngs		
		0	276 (23.0)
		1	781 (65.0)
		2	112 (9.3)
		3+	33 (2.8)
	Vigorous physical activity		
		Never or occasionally	1023 (85.1)
		Once or twice per week	130 (10.8)
		Three or more times a week	49 (4.1)
	Watch television		
		Less than 1 hour	199 (16.6)
		1 hour but less than 3 hours	672 (55.9)
		3 hours but less than 5 hours	189 (15.7)
		5 hours or more	142 (11.8)
	Fuel for cooking		
		Electricity	84 (7.0)
		Gas	1078 (89.7)
		Open fires	7 (0.6)
		Others	33 (2.8)
	Paracetamol use in the first 12 months of life		
		Yes	1049 (87.3)
		No	153 (12.7)
	Paracetamol use in the past 12 months		
		Never	267 (22.2)
		at least once a year	767 (63.8)
		at least once per month	168 (14.0)
	Antibiotics in the first 12 months		
		Yes	876 (72.9)
		No	326 (27.1)
	Trucks pass through the street		
		Never	512 (42.6)
		Seldom	324 (27.0)
		Frequently through the day	279 (23.2)
		Almost the whole day	87 (7.2)
	Breast feeding		
		Yes	1.152 (95.8)
		No	50 (4.2)
	Cat or dog in home in the first year of life		
		Yes	333 (27.7)
		No	869 (72.3)
	Cat or dog in home in the past 12 months		
		Yes	335 (27.9)
		No	967 (72.1)
	Contact with farm animals in the first year of life		
		Yes	77 (6.4)
		No	1125 (93.6)
	Mother regular contact with farm animals		
		Yes	110 (9.2)
		No	1092 (90.9)
	Living in urban communes		
		Yes	768 (63.9)
		No	434 (36.1)
	Mother smoke		
		Yes	6 (0.5)
		No	1196 (99.5)
	Mother smoke during child’s first year of life		
		Yes	7 (0.6)
		No	1195 (99.4)
	Father smoke		
		Yes	629 (52.3)
		No	573 (47.7)
	Father smoke during mother’s pregnancy		
		Yes	684 (56.9)
		No	518 (43.1)
	Smoker(s) in household		
		Yes	685 (57.0)
		No	517 (43.0)

### Allergic characteristics of participants

Table 2 lists the numbers and proportions of “yes” answers to each the International Study of Asthma and Allergies in Childhood (ISAAC) question^25^ related to wheezing, allergic rhinitis, and eczema symptoms. The children’s caregivers did not answer a question about “hay fever” because they did not think there was hay fever in Vietnam. The proportions of children with asthma, rhinoconjunctivitis, and eczema were 5.1% (95% confidence interval [CI] 3.9–6.5%), 11.5% (95% CI 9.7–13.4%), and 6.7% (95% CI 5.3–8.2%), respectively.

**Table 2:** Asthma, rhinoconjunctivitis, and eczema among the children who participated in the six-year follow-up survey.

Variable	Number	Prevalence (%)
Wheeze ever	136	11.3
Wheeze in the past 12 months	61	5.1
4 or more attacks of wheeze in the past 12 months	12	1.0
Sleep disturbance from wheeze, 1 or more nights a week in the past 12 months	4	0.3
Speech limited by wheeze in the past 12 months	4	0.3
Asthma ever	38	3.2
Wheeze during or after exercise in the past 12 months	18	1.5
Night cough in the past 12 months	156	13.0
**Asthma**	**61**	**5.1**
Nose symptoms ever	573	47.7
Nose symptoms in the past 12 months	409	34.0
Nose and eye symptoms in the past 12 months	138	11.5
Nose symptoms affecting activities a lot in the past 12 months	10	0.8
Hay fever ever	―	―
**Rhinoconjunctivitis**	**138**	**11.5**
Rash ever	126	10.5
Rash in the past 12 months	94	7.8
Flexural rash	80	6.7
Complete clearance of rash in the past 12 months	68	5.7
Sleep disturbance from rash, 1 or more nights a week in the past 12 months	4	0.3
Eczema ever	19	1.6
**Eczema**	**80**	**6.7**
Number of participants	1202	

### Risk factors for asthma at age 6

Children born with low birthweight had some risks for asthma at age 6 (adjusted odds ratio [aOR] 2.93, 95% CI 0.83–10.40) (Table 3), but preterm born and light-for-dates children had no association with asthma at age 6 (aOR 1.14 [0.26–4.97] and 0.81 [0.31–2.08], respectively, Table 4, sensitivity analysis). Heavy birthweight (>4000 g referenced to 2500–4000 g) (Table 4), high current body weight (≥25 kg referenced to <25 kg) (in Supplementary Table 1), and high catch-up (referenced to non-high catch-up) (Table 4) were not associated with asthma.

**Table 3:** Risk factor analysis of asthma, rhinoconjunctivitis, and eczema.

Characteristics (n = 1202)			Number (%)	Asthma, aOR^a^ (95% CI)	Rhinoconjunctivis aOR^a^ (95% CI)	Eczema, aOR^a^ (95% CI)
**Demographics**						
	Sex	Male	610 (50.8)	1.05 (0.62–1.78)	1.11 (0.78–1.60)	1.60 (0.99–2.58)
		Female	592 (49.3)	1.00	1.00	1.00
**Perinatal information**						
	Birthweight					
		<2500 g	27 (2.3)	2.93 (0.83–10.40)	1.56 (0.52–4.68)	5.12 (1.92–13.64)
		>=2500 g	1175 (97.8)	1.00	1.00	1.00
**Family history**						
(n = 1201)	Maternal history of asthma and allergic diseases					
		Yes	96 (8.0)	2.38 (1.14–4.93)	1.79 (1.01–3.18)	2.31 (1.21–4.41)
		No	1105 (92.0)	1.00	1.00	1.00
(n = 1191)	Paternal history of asthma and allergic diseases					
		Yes	47 (4.0)	0.74 (0.17–3.20)	2.38 (1.13–5.04)	3.48 (1.56–7.77)
		No	1144 (96.1)	1.00	1.00	1.00
**Socioeconomic status**						
(n = 1201)	Maternal education level					
		College or university	248 (20.6)	2.51 (0.77–8.16)	0.92 (0.49–1.74)	0.90 (0.39–2.09)
		High school	399 (33.2)	2.37 (0.77–7.27)	0.71 (0.39–1.31)	1.01 (0.47–2.13)
		Secondary school	403 (33.5)	1.88 (0.62–5.72)	0.66 (0.36–1.22)	0.78 (0.37–1.64)
		No or primary school	152 (12.7)	1.00	1.00	1.00
**Environmental status**						
	Number of siblings					
		3+	33 (2.8)	2.34 (0.47–11.60)	NA^b^	1.40 (0.36–5.48)
		2	112 (9.3)	1.78 (0.61–5.21)	0.40 (0.14–1.08)	1.24 (0.51–3.02)
		1	781 (65.0)	1.54 (0.75–3.14)	1.51 (0.95–2.38)	1.13 (0.63–2.02)
		0	276 (23.0)	1.00	1.00	1.00
	Living in urban communes					
		Yes	768 (63.9)	1.20 (0.66–2.16)	0.88 (0.60–1.30)	1.43 (0.84–2.45)
		No	434 (36.1)	1.00	1.00	1.00
	Smoker(s) in household					
		Yes	685 (57.0)	0.89 (0.52–1.52)	1.05 (0.72–1.52)	1.46 (0.89–2.39)
		No	517 (43.0)	1.00	1.00	1.00

^a^OR, adjusted odds ratio; 95% CI, 95% confidence interval; NA, not applicable.

^a^A model incorporated sex, low birthweight, maternal and paternal asthma and allergic disease history, maternal educational level, number of siblings, living in urban area, and environmental tobacco smoke exposure.

^b^It is not applicable because no children with current rhinoconjunctivitis had 3 or more siblings.

**Table 4:** Odds ratios and adjusted odds ratios of asthma, rhinoconjunctivitis and eczema symptoms for birthweight, gestational age, and light-for-dates of the participating children.

Characteristics	N	Cases	%	Asthma symptoms crude OR (95% CI)	Asthma symptoms adjusted OR^a^ (95% CI)	Cases	%	Rhinoconjuncti-vitis symptoms crude OR (95% CI)	Rhinoconjuncti-vitis symptoms adjusted OR^a^ (95% CI)	Cases	%	Eczema symptoms crude OR (95% CI)	Eczema symptoms adjusted OR^a^ (95% CI)
**Birthweight**													
<2500 g	27	3	11.1	2.41 (0.70–8.23)	2.93 (0.83–10.40)	4	14.8	1.35 (0.46–3.97)	1.56 (0.52–4.69)	6	22.2	4.25 (1.67–10.85)	5.13 (1.93–13.62)
<= 2500 g	1175	58	4.9	1.00	1.00	134	11.4	1.00	1.00	74	6.3	1.00	1.00
**Birthweight**													
<2500 g	27	3	11.1	2.42 (0.71–8.29)	2.95 (0.83–10.46)	4	14.8	1.33 (0.45–3.90)	1.55 (0.51–4.64)	6	22.2	4.44 (1.74–11.36)	5.31 (2.00–14.12)
2500–4000 g	1141	56	4.8	1.00	1.00	132	11.6	1.00	1.00	69	6.1	1.00	1.00
>4000 g	34	2	5.8	1.21 (0.28–5.18)	1.24 (0.28–5.38)	2	5.9	0.48 (0.11–2.02)	0.50 (0.11–2.15)	5	14.7	2.68 (1.01–7.14)	2.54 (0.92–6.98)
**Gestational age**													
<37 weeks (preterm)	36	2	5.6	1.10 (0.26–4.70)	1.14 (0.26–4.97)	5	13.9	1.25 (0.48–3.28)	1.45 (0.54–3.90)	2	5.6	0.82 (0.19–3.48)	0.84 (0.20–3.63)
>= 37 weeks	1166	59	5.1	1.00	1.00	133	11.4	1.00	1.00	78	6.7	1.00	1.00
**Light-for-dates**													
Light-for-dates	129	6	3.9	0.73 (0.29–1.86)	0.81 (0.31–2.08)	10	7.8	0.62 (0.32–1.21)	0.66 (0.33–1.30)	9	7.0	1.06 (0.52–2.17)	1.15 (0.55–2.40)
Appropriate/heavy-for-dates	1073	56	5.2	1.00	1.00	128	11.9	1.00	1.00	71	6.6	1.00	1.00
**High catch-up in weight**													
High catch-up	120	7	5.8	1.18 (0.52–2.65)	1.25 (0.55–2.84)	15	12.5	1.11 (0.63–1.97)	1.09 (0.61–1.96)	7	6.8	0.86 (0.38–1.90)	0.83 (0.37–1.87)
Non high catch-up	1082	54	5.0	1.00	1.00	123	11.4	1.00	1.00	73	5.8	1.00	1.00

OR, odds ratio; 95% CI, 95% confidence interval. ^a^A model incorporated sex, one of anthropometric characteristics (Birthweight < 2500 g/ >= 2500 g, birthweight < 2500 g/ 2500–4000 g/ > 4000 g, preterm born, light-for-dates, or high catch-up in weight), maternal and paternal asthma and allergic disease history, maternal educational level, number of siblings, living in urban area, and environmental tobacco smoke exposure.

Maternal, but not paternal, history of asthma and allergic diseases independently increased the child’s odds of having asthma (aOR 2.38, 95% CI 1.14–4.93) (Table 3).

### Risk factors for rhinoconjunctivitis at 6 years of age

Any parameters of anthropometry at birth, as well as sex, maternal education level, number of siblings, living in urban areas, and environmental tobacco smoke (ETS) exposure were not related to rhinoconjunctivitis at age 6 (Tables 3 and 4).

Maternal and paternal history of asthma and allergic diseases independently increased the child’s odds of having rhinoconjunctivitis (aOR 1.79, 95% CI 1.01–3.18 and aOR 2.38, 95% CI 1.13–5.04, respectively) (Table 3).

### Risk factors for eczema at 6 years of age

Low birthweight was strongly associated with eczema (crude odds ratio [OR] 4.25, 95% CI 1.67–10.85) and the OR was slightly increased after adjusting for other variables in the model (aOR 5.12, 95% CI 1.92–13.64) (Supplementary Table 1 and Table 3); however, other anthropometric factors, including high birthweight, preterm born, light-for-dates, and high catch-up were not associated with eczema (Table 4). The aOR of eczema for low birthweight only in full term born children (n = 1166) was 8.42 (95% CI 2.44–29.05).

Maternal and paternal history of asthma and allergic diseases independently increased the child’s odds of having eczema (aOR 2.31, 95% CI 1.21–4.41 and aOR 3.48, 95% CI 1.56–7.77, respectively) (Table 3). There was no association of eczema at age of 6 with sex, maternal education level, number of siblings, living in urban areas, or ETS exposure (Table 3).

## Discussion

In Nha Trang City, South-Central Vietnam, asthma, rhinoconjunctivitis, and eczema among a cohort of 6-year-old children were 5.1%, 11.5%, and 6.7%, respectively. Low birthweight, maternal and paternal history of asthma and allergic diseases were independently associated with an increased risk of eczema at 6 years of age. Low birthweight tended to increase asthma but the association was not significant. This study demonstrated the effect of anthropometry at birth on asthma, rhinoconjunctivitis, and eczema in childhood, with consideration of social, familial, and environmental factors in Vietnam.

### Prevalence of asthma, rhinoconjunctivitis, and eczema at age 6 in Vietnam

The ISAAC Phase Three study between 2002 and 2003 revealed the prevalence of asthma, rhinoconjunctivitis, and eczema among children aged 6–7 years from 66 centers in 37 countries^1^. The prevalence of asthma in Canada, UK, and Australia was 18.2%, 20.9%, and 20.0%, respectively, and in Hong Kong, Indonesia, Japan, Malaysia, Singapore, South Korea, Taiwan, and Thailand it was 9.4%, 2.8%, 18.2%, 5.8%, 10.2%, 5.8%, 9.8%, and 11.9%, respectively^1^. In the birth cohort study, the prevalence of asthma was lower than in the countries where the majority are people of European background (Canada, UK, and Australia) and relative low even compared with the eight Asian countries listed above. The prevalence of rhinoconjunctivitis in Canada, UK, and Australia was 10.8%, 10.1%, and 12.9%, respectively, and 17.7%, 3.6%, 10.6%, 4.8%, 8.7%, 8.7%, 24.2%, and 10.4%, respectively, in the eight Asian counties^1^, which is similar to the prevalence found in this study. The prevalence of eczema in Canada, UK, and Australia was 12.0%, 16.0%, and 17.1%, respectively, and in Hong Kong, Malaysia, Singapore, South Korea, Taiwan, and Thailand it was 4.6%, 12.6%, 8.9%, 11.3%, 6.7%, and 16.7%, respectively^1^, which, in general, is higher than the prevalence found in the birth cohort study. The ISAAC Phase Three study was conducted 15 years before the current study, so the prevalence of these symptoms might have increased in many of these countries. A rise in the prevalence was seen between the Phase One and the Phase Three ISAAC studies. In 1999 in Hanoi, Vietnam, the prevalence of asthma and rhinoconjunctivitis, and “ever had eczema” among 5–11-year-old children was 14.9%, 10.7%, and 3.3%, respectively^22^. In 2001 in Ho Chi Minh City, Vietnam, the prevalence of asthma, rhinoconjunctivitis, and eczema among children aged 6–7 years was 17.8%, 10.1%, and 4.7%, respectively^25^. The data from these two cities in Vietnam were compiled 15 years or more before the birth cohort study. In the present study, the prevalence of asthma was much lower than in the previous studies, and the prevalence of rhinoconjunctivitis and eczema was similar. Behavior pattern or level of air pollution in these two big cities might be different from those in Nha Trang and may have caused the difference in the prevalence of asthma.

### Low birthweight and asthma, rhinoconjunctivitis, and eczema

A novel finding in this study was the association between low birthweight and higher prevalence of eczema at the age of 6 years. Previous studies found contradictory or no association between them^13–16^. Birthweight and gestational age should be highly correlated but we found no positive association between preterm born and eczema. The effect of low birthweight on eczema was still significant among only full-term-born children. A previous study found a positive association between fetal growth and childhood atopic eczema, hypothesizing that immature development of their immune system during fetal growth may have disturbed sensitization or Th1/Th2 balance and caused atopic eczema after the birth^13,26^. However, the mechanism involved in the occurrence of eczema is not fully known. The reason for the contrary result in the birth cohort study is also unknown, but may be related to immunogenetics, diets, allergens, or the tropical climate in Vietnam, because most of the previous studies were conducted among people of European background and in temperate or cool-temperate regions. Factors influencing expression of atopic eczema in tropical regions have been reported; for example, sweating, drying out the skin by frequently washing, and incomplete washing out of soap can aggravate the symptoms, whereas ultraviolet exposure is likely to be a protective factor^27^. Such factors may have influenced our results, even though the mechanism is likely to be multifactorial.

Children born with low birthweight tended to have a higher prevalence of asthma at the age of 6 years after adjusting several socioeconomic and familial factors. Some previous studies have shown that low birthweight was a significant risk factor for subsequent wheeze or asthma in childhood^5,8,28^, while others have reported no correlation^29,30^. There may be no or only weak association between low birthweight and asthma in childhood, or the effect may be small because of the small number of children born with low birthweight (n = 27) and their relative heavy birthweights (lowest birthweight was 1620 g) in the current study. High birthweight (>4000 g), high weight at age 6 years, and high catch-up from lowest tertile birthweight to highest tertile weight at age 6 years were not associated with current asthma. Some studies have found positive association between these factors^10,11,31^, while others have not^8^.

There was no association between low birthweight and rhinoconjunctivitis in the current study as most of the previous studies^15,18,19^, while one study showed that low birthweight was positively associated with allergic rhinitis and positive skin prick test at the age of 2 years^20^.

### Limitations

In this study, we used only the information obtained from interviews using the questionnaire for current symptoms, without any objective information such as doctor-diagnosed diseases, skin prick test, serum immunoglobulin E level measurement, or spirometry test for asthma. The information could be inaccurate because of respondents’ lapse of memory or misdiagnosis of the symptoms. Hence the results could result in under or overestimation of the outcomes, however, the ISAAC questionnaire used in the birth cohort study has been validated extensively in different populations and we believe it can be a reliable method for this epidemiological survey. Also, the accuracy of the information is likely to be similar for children with and without low birthweight when comparing the effect of low birthweight on asthma, rhinoconjunctivitis, and eczema. Further studies using the more objective examinations are needed to obtain more precise information and to know the mechanism that caused the result.

## Conclusion

We determined the prevalence of asthma, rhinoconjunctivitis, and eczema in children at the age of 6 years in Nha Trang City, South-Central Vietnam. The study demonstrated that low birthweight is an important risk factor for eczema in young children, and is independent of socioeconomic and environmental factors, and other characteristics at birth.

## Methods

### Study design and participants

This study is part of a birth cohort study in Nha Trang City, South-Central Vietnam. For the birth cohort study, women aged ≥17 years and residing in 16 target communes in Nha Trang who delivered a single child at a provincial hospital without severe complications between May 2009 and May 2010 were recruited, together with their newborn babies. Babies’ body weight at birth and clinical information were collected using a structured questionnaire and from medical charts by two trained research nurses under the supervision of a research clinician. Detailed information of the setting is described elsewhere^32,33^.

From March to August in 2016, we conducted a follow-up survey of the birth cohort children living in the catchment area at the time of the survey. The children and their caregivers were invited to the commune health center in a commune where they live. During their visit anthropometric measurements were made, and interviews to obtain epidemiological and familial information, past and present medical history, and allergic status were conducted by trained medical staff in the commune health center using a structured questionnaire. Written informed consent was obtained from a parent and/or legal guardian for the study participants before participation.

### Asthma, rhinoconjunctivitis, and eczema

Questions of asthma, rhinoconjunctivitis, and eczema were adapted from the International Study of Asthma and Allergies in Childhood (ISAAC), Phase Three Core and Environmental Questionnaire for 6–7-year-old children^25^. Vietnamese version of the ISAAC questionnaire was developed through forward-backward translations. We estimated asthma based on positive answers to the question: “Has your child had wheezing or whistling in the chest in the past 12 months?” Current allergic rhinoconjunctivitis were estimated based on positive answers to both these questions: “In the past 12 months, has your child had a problem with sneezing or a runny or blocked nose when he/she did not have a cold or the flu?” and if yes, “In the past 12 months, has this nose problem been accompanied by itchy watery eyes?” Current eczema was estimated based on positive answers to two questions: “Has your child had this itchy rash at any time in the past 12 months?” and, “Has this itchy rash at any time affected any of the following places: the folds of the elbows; behind the knees; in front of the ankles; under the buttocks; or around the neck, ears, or eyes?” These questions were preceded by the question “Has your child ever had a skin rash which was coming and going for at least 6 months?”^1^.

### Other variables

Maternal (paternal) history of asthma and allergic diseases was defined as maternal (paternal) history of asthma, atopic eczema, or rhinitis. Maternal educational level was categorized into four groups, based on the educational system in Vietnam, namely none–primary, secondary, high school, and college or university. ETS exposure was defined as presence of smoker(s) in the household. This information was obtained from the interview. Residential areas where the children lived at the time of the follow-up survey were defined as urban or rural according to the Vietnam government administrative categorization. Births were considered preterm if they occurred at less than 37 gestational weeks, and low birthweight was defined as less than 2500 grams, following the guidelines in the International Classification of Diseases-10: version 2010^34^. “Light-for-dates” was defined as birthweight below the 10th percentile^34^, identified from a diagram of birthweight percentiles by gestational weeks created based on data collected from the enrollments of this birth cohort study^35^. “High catch-up” was defined as birthweight in the lowest tertile and current body weight in the highest tertile.

### Sample size

We assumed the true proportion of current asthma, rhinoconjunctivitis, or eczema to be 0.5 to have the largest sample size and needed the estimate to be within 0.03 of the true value (precision) with 95% CI. Then, the sample size was calculated to be 1067 or more.

### Statistical analysis

Baseline characteristics, and numbers and proportions of the children in the follow-up survey who had asthma, rhinoconjunctivitis, and eczema as defined above, were described by simple tabulations. ORs of asthma, rhinoconjunctivitis, and eczema for each demographic, perinatal, familial, and environmental factor were estimated using logistic regression. The following potential confounders were identified *a priori* and were included in the multivariable model to estimate aORs of asthma, rhinoconjunctivitis, or eczema for low birthweight (birthweight <2500 g referenced to ≥2500 g): sex, paternal and maternal history of asthma and allergic diseases, maternal educational level, number of siblings (0, 1, 2, 3, or more), living in urban area, and ETS exposure (original model). Sensitivity analyses using other anthropometry or anthropometry related parameters at birth instead of low birthweight (<2500 g, ≥2500 g) were conducted as follows and the results were summarized in a separate table. The same model with a different categorization of birthweight (birthweight <2500 g, 2500–4000 g, ≥4000 g) was used for asthma, rhinoconjunctivitis, and eczema to determine the effect of high birthweight. The aORs for anthropometry parameters, including preterm birth, light-for-dates, and high catch-up, were estimated using a model with the variables defined above, except for birthweight. We calculated aORs for low birthweight only in full-term born children to remove the effect of prematurity on birthweight. P values < 0.05 were considered statistically significant. Statistical analyses were conducted using STATA version 14.0 (StataCorp LLC, TX, USA).

### Ethical consideration

This study was approved by the Ethical Committees of the Institute of Tropical Medicine, Nagasaki University, Japan (approval number 151203147) and the National Institute of Hygiene and Epidemiology, Vietnam (IRB-VN01057-20/2015) and this study was conducted in accordance with the Declaration of Helsinki in 2013^36^.

## Supplementary information

Table S1

## Data Availability

The interested readers can obtain the data by writing to the corresponding author of this manuscript.
