# Supplementary material for: Asthma, Rhinoconjunctivitis, Eczema, and the Association with Perinatal Anthropometric Factors in Vietnamese Children
Source: Sci Rep. 2019 Feb 25;9:2655. doi: 10.1038/s41598-019-39658-5 (PMC6389945; doi:10.1038/s41598-019-39658-5)
Supplement: Supplementary file 1 — Table S1 [file 41598_2019_39658_MOESM1_ESM.pdf]

Title

Asthma, Rhinoconjunctivitis, Eczema, and the Association with Perinatal Anthropometric Factors in Vietnamese Children

Michiko Toizumi, Masahiro Hashizume, Hien Anh T. Nguyen, Michio Yasunami, Noriko Kitamura, Chihiro Iwasaki, Mizuki Takegata, Hiroyuki Moriuchi, Duc Anh Dang, Koya Ariyoshi, and Lay-Myint Yoshida

Table S1. Crude odds ratios of asthma, rhinoconjunctivitis, and eczema for each characteristic.

Characteristics (n=1202)			Total number (%)	Asthma crude OR (95% CI)	Rhinoconjunctivitis crude OR (95% CI)	Eczema crude OR (95% CI)
**Demographics**						
	Sex	Male	610 (50.8)	1.00 (0.60-1.68)	1.10 (0.77-1.57)	1.58 (0.99-2.52)
		Female	592 (49.3)	1.00	1.00	1.00
	Body weight					
		25kg or more	568 (47.3)	1.08 (0.65-1.82)	1.25 (0.88-1.78)	1.07 (0.68-1.68)
		less than 25kg	634 (52.8)	1.00	1.00	1.00
**Perinatal information**						
	Mode of delivery					
		Vaginal	688 (57.2)	1.00	1.00	1.00
		Caesarean section	514 (42.8)	1.41 (0.84-2.36)	1.14 (0.80-1.63)	0.89 (0.56-1.41)
	Birthweight					
		>=2500g	1175 (97.8)	1.00	1.00	1.00
		<2500g	27 (2.3)	2.41 (0.70-8.23)	1.35 (0.46-3.97)	4.25 (1.67-10.85)
**Family history**						
	Mother's age at child's birth					
		24 years or less	312 (26.0)	1.00	1.00	1.00
		25-34 years	728 (60.6)	1.24 (0.66-2.31)	1.64 (1.05-2.57)	1.92 (1.03-3.56)
		35 years or more	162 (13.5)	0.96 (0.38-2.43)	0.92 (0.46-1.83)	1.68 (0.73-3.83)
(n=1201)	Maternal history of asthma and allergic diseases					
		Yes	96 (8.0)	2.40 (1.18-4.90)	1.75 (1.00-3.05)	2.69 (1.45-4.99)
		No	1105 (92.0)	1.00	1.00	1.00
(n=1201)	Maternal history of asthma					
		Yes	12 (1.0)	3.83 (0.82-17.88)	1.55 (0.34-7.14)	1.28 (0.16-10.02)
		No	1189 (99.0)	1.00	1.00	1.00
(n=1201)	Maternal history of atopic eczema					
		Yes	13 (1.1)	5.84 (1.57-21.81)	0.64 (0.08-4.95)	4.33 (1.17-16.05)
		No	1188 (98.9)	1.00	1.00	1.00
(n=1201)	Maternal history of rhinitis					
		Yes	76 (6.3)	1.34 (0.52-3.46)	2.00 (1.10-3.63)	2.59 (1.31-5.13)
		No	1125 (93.7)	1.00	1.00	1.00
(n=1191)	Paternal history of asthma and allergic diseases					
		Yes	47 (4.0)	0.83 (0.20-3.52)	2.15 (1.04-4.42)	3.58 (1.67-7.69)
		No	1144 (96.1)	1.00	1.00	1.00
(n=1191)	Paternal history of asthma					

	Yes	9 (0.8)	2.38 (0.29-19.34)	0.95 (0.12-7.68)	NA
	No	1182 (99.2)	1.00	1.00	1.00
(n=1191)	Paternal history of atopic eczema				
	Yes	5 (0.4)	4.78 (0.53-43.40)	1.91 (0.21-17.25)	3.50 (0.39-31.72)
	No	1186 (99.6)	1.00	1.00	1.00
(n=1191)	Paternal history of rhinitis				
	Yes	33 (2.8)	NA	2.53 (1.12-5.73)	4.83 (2.10-11.08)
	No	1158 (97.2)	1.00	1.00	1.00
**Socioeconomic status**					
(n=1157)	Wealth level				
	Low	386 (33.4)	1.00	1.00	1.00
	Middle	386 (33.4)	1.00 (0.51-1.95)	1.16 (0.75-1.81)	1.90 (1.03-3.49)
	High	385 (33.3)	1.18 (0.62-2.25)	1.14 (0.73-1.77)	1.97 (1.07-3.61)
(n=1201)	Maternal education level				
	No or primary school	152 (12.7)	1.00	1.00	1.00
	Secondary school	403 (33.5)	1.45 (0.53-3.97)	0.84 (0.47-1.52)	0.74 (0.36-1.52)
	High school	399 (33.2)	1.72 (0.64-4.62)	0.90 (0.50-1.61)	0.88 (0.44-1.78)
	College or university	248 (20.6)	1.89 (0.67-5.32)	1.26 (0.69-2.32)	0.80 (0.37-1.75)
**Environmental status**					
	Number of siblings				
	0	276 (23.0)	1.00	1.00	1.00
	1	781 (65.0)	1.55 (0.77-3.13)	1.53 (0.97-2.40)	1.06 (0.60-1.88)
	2	112 (9.3)	1.51 (0.53-4.25)	0.45 (0.17-1.20)	1.33 (0.57-3.08)
	3+	33 (2.8)	1.72 (0.36-8.19)	NA	1.52 (0.42-5.50)
	Vigorous physical activity				
	Never or occasionally	1023 (85.1)	1.00	1.00	1.00
	Once or twice per week	130 (10.8)	0.61 (0.22-1.70)	0.48 (0.23-1.01)	0.51 (0.20-1.29)
	Three or more times a week	49 (4.1)	2.66 (1.08-6.54)	1.22 (0.54-2.77)	0.27 (0.04-1.96)
	Watch television				
	Less than 1 hour	199 (16.6)	1.00	1.00	1.00
	1hour but less than 3 hours	672 (55.9)	1.64 (0.72-3.74)	0.86 (0.53-1.41)	0.96 (0.53-1.76)
	3 hours but less than 5 hours	189 (15.7)	0.90 (0.30-2.73)	0.91 (0.49-1.70)	0.40 (0.15-1.06)
	5 hours or more	142 (11.8)	2.08 (0.77-5.60)	1.34 (0.72-2.49)	0.93 (0.40-2.13)
	Fuel for cooking				
	Electricity	84 (7.0)	1.00	1.00	1.00

Gas	1078 (89.7)	0.90 (0.21-3.84)	1.67 (0.71-3.92)	1.43 (0.51-4.02)
Open fires	7 (0.6)	NA	2.17 (0.22-21.06)	NA
Others	33 (2.8)	NA	4.16 (1.32-13.14)	2.76 (0.65-11.76)
Paracetamol use in the first 12 months of life				
Yes	1049 (87.3)	2.93 (0.91-9.46)	1.22 (0.69-2.15)	1.02 (0.52-2.03)
No	153 (12.7)	1.00	1.00	1.00
Paracetamol use in the past 12 months				
Never	267 (22.2)	1.00	1.00	1.00
at least once a year	767 (63.8)	3.42 (1.35-8.69)	1.80 (1.08-3.01)	0.98 (0.55-1.74)
at least once per month	168 (14.0)	2.97 (0.98-9.01)	2.39 (1.28-4.47)	1.44 (0.70-2.97)
Antibiotics in the first 12 months				
Yes	876 (72.9)	2.56 (1.20-5.44)	1.46 (0.95-2.24)	1.05 (0.63-1.76)
No	326 (27.1)	1.00	1.00	1.00
Trucks pass through the street				
Never	512 (42.6)	1.00	1.00	1.00
Seldom	324 (27.0)	1.06 (0.57-1.95)	0.90 (0.59-1.39)	0.96 (0.56-1.67)
Frequently through the day	279 (23.2)	0.74 (0.36-1.51)	0.78 (0.49-1.25)	0.75 (0.40-1.40)
Almost the whole day	87 (7.2)	1.10 (0.41-2.93)	0.81 (0.39-1.69)	1.16 (0.50-2.69)
Breast feeding				
Yes	1.152 (95.8)	0.83 (0.25-2.75)	2.08 (0.64-6.77)	0.81 (0.28-2.32)
No	50 (4.2)	1.00	1.00	1.00
Cat or dog in home in the first year of life				
Yes	333 (27.7)	1.01 (0.57-1.79)	1.12 (0.76-1.65)	0.69 (0.40-1.19)
No	869 (72.3)	1.00	1.00	1.00
Cat or dog in home in the past 12 months				
Yes	335 (27.9)	1.00 (0.56-1.78)	1.11 (0.75-1.63)	1.12 (0.68-1.83)
No	967 (72.1)	1.00	1.00	1.00
Contact with farm animals in the first year of life				
Yes	77 (6.4)	1.03 (0.36-2.91)	0.64 (0.27-1.49)	0.55 (0.17-1.79)
No	1125 (93.6)	1.00	1.00	1.00
Mother regular contact with farm animals				
Yes	110 (9.2)	0.32 (0.08-1.35)	1.14 (0.63-2.06)	1.46 (0.73-2.92)
No	1092 (90.9)	1.00	1.00	1.00
Living in urban communes				
Yes	768 (63.9)	1.37 (0.78-2.41)	1.03 (0.71-1.49)	1.53 (0.92-2.54)
No	434 (36.1)	1.00	1.00	1.00
Mother smoke				

Yes	6 (0.5)	3.79 (0.44-32.92)	1.55 (0.18-13.33)	2.83 (0.33-24.50)
No	1196 (99.5)	1.00	1.00	1.00
Mother smoke during child's first year of life				
Yes	7 (0.6)	3.50 (0.37-26.61)	1.29 (0.15-10.77)	NA
No	1195 (99.4)	1.00	1.00	1.00
Father smoke				
Yes	629 (52.3)	0.71 (0.42-1.19)	0.93 (0.65-1.33)	1.32 (0.83-2.09)
No	573 (47.7)	1.00	1.00	1.00
Father smoke during mother's pregnancy				
Yes	684 (56.9)	0.67 (0.35-1.29)	0.64 (0.45-0.91)	1.52 (0.94-2.46)
No	518 (43.1)	1.00	1.00	1.00
Smoker(s) in household				
Yes	685 (57.0)	0.88 (0.53-1.48)	1.05 (0.73-1.50)	1.52 (0.94-2.46)
No	517 (43.0)	1.00	1.00	1.00

OR, odds ratio; 95% CI, 95% confidence interval
